# Unfolding the values of work – therapists´ experience of addressing the return to work process in occupational rehabilitation based on Acceptance and Commitment Therapy

**DOI:** 10.1186/s12913-018-3035-8

**Published:** 2018-04-27

**Authors:** Nina E. Klevanger, Marius S. Fimland, Roar Johnsen, Marit B. Rise

**Affiliations:** 1 Department of Public Health and Nursing, Faculty of Medicine and Health Sciences, Norwegian University of Science and Technology, Trondheim, Norway; 2 Department of Neuromedicine and Movement Science, Faculty of Medicine and Health Sciences, Norwegian University of Science and Technology, Trondheim, Norway; 3 Unicare Helsefort Rehabilitation Centre, Rissa, Norway; 4 Department of Mental Health, Faculty of Medicine and Health Sciences, Norwegian University of Science and Technology, Trondheim, Norway

**Keywords:** Sick leave, Absenteeism, Musculoskeletal diseases, Mental health, Occupational health, Rehabilitation, Cognitive behaviour therapy, Return to work, Patient participation

## Abstract

**Background:**

Facilitating return to work can be challenging due to the complexity of work disability. Few studies have examined rehabilitation programs based on Acceptance and Commitment Therapy that intend to support return to work, and none have investigated therapists’ experience with providing such programs. The aim of this study was therefore to explore therapists’ experience of addressing the return to work process in an inpatient occupational rehabilitation program based on Acceptance and Commitment Therapy.

**Methods:**

This was a qualitative interview study supported by participant observation. Therapists were interviewed regarding their experiences with addressing return to work in an inpatient occupational rehabilitation program based on Acceptance and Commitment Therapy. In addition, the rehabilitation program was investigated through participant observation. The interviews were analysed according to Interpretative Phenomenological Analysis and informed by an analysis of field notes from the participant observation.

**Results:**

Acceptance and Commitment Therapy was experienced as a meaningful approach to facilitate return to work, as it allowed therapists to address all relevant aspects of the individual participant’s life that might influence work participation. The therapists’ twofold goal was to support participants in building both a meaningful life and sustainable work participation. To do so, they attempted to instil long-term and interrelated processes concerning ownership, causes of sick leave, relation to expectations, the values of work, and the scope of agency.

**Conclusion:**

Unfolding values connected to work participation might reconcile the tension between work and family life by integrating work with other areas of life. Providing work participation with personal meaning also seems especially commensurable with a context where economy presents a poor incentive for return to work. Therapists should, however, be attentive to the need to secure the prominence of return to work by relating participants’ chosen themes explicitly to their return to work process. Therapists should also be aware of the dilemma that may arise when they attempt to refrain from providing advice while simultaneously encouraging actions they consider appropriate to facilitate sustainable work participation. In addition, having an individual-oriented approach to occupational rehabilitation may obscure the extent to which return to work is a multi-stakeholder process.

## Background

Over the last 20 years, a paradigm shift is said to have taken place within return to work (RTW) research and evidence-informed practice, going from unidisciplinary approaches (e.g. biomedical), to multi- and transdisciplinary biopsychosocial models [1]. Among the factors suggested to influence RTW independent of injury/illness are gender, age, education, socioeconomic status, level of injury/illness, self-efficacy/expectations for recovery and RTW, work demands, RTW coordination, and multidisciplinary interventions [2]. Encounters between patients and health/RTW professionals have also been identified as important [3–6] as well as the roles, beliefs and perceptions of various stakeholders, the organizational environment, and the compensation and health care systems [7]. As it is now recognised that long-term work disability can be upheld by factors other than the triggering injury or disability, it has been proposed that rehabilitation programs need to encompass the complexity and multi-causality of work disability [8–10].

In Norway, musculoskeletal and mental health diagnoses account for approximately 60% of sick leave days [11]. To prevent work disability, different legislative, health, safety and environmental acts have been implemented [12, 13], and a variety of rehabilitation programs are provided within tertiary institutional care. Between 2010 and 2016, Hysnes Rehabilitation Centre provided 3.5-weeks of inpatient occupational rehabilitation to participants on sick leave due to musculoskeletal pain, common mental disorders and/or other non-specific disorders [14, 15]. The program was multidisciplinary and consisted of physical training, work-related problem solving and psychological treatment provided in individual sessions and to groups with different diagnoses. Acceptance and Commitment Therapy (ACT) was the prevailing approach for all components of the program. ACT is part of the third wave of cognitive behavioural therapies, and uses acceptance-, mindfulness-, commitment- and behaviour-change processes to increase psychological flexibility [16]. Psychological flexibility is described as “the ability to contact the present moment more fully as a conscious human being, and to change or persist in behaviour when doing so serves valued ends” [17]. Clarifying personal values, the long-termed, desired qualities of life, is a key ingredient of the therapy [17]. Unlike goals, values can never be obtained; instead, they provide a direction for committed action. As ACT seeks to develop broad and generally applicable skills [18], it has been used to treat various conditions, including pain and mental disorders [19–22]. While few studies have explored the use of ACT in combination with RTW rehabilitation, some have investigated the effect of ACT-based treatment on work participation [23–27]. In addition, a previous study found that participants, after taking part in ACT-based occupational rehabilitation, perceived RTW as part of a long and complex process that had to be balanced with other desired values [28]. No studies have so far investigated therapists’ experiences with providing RTW rehabilitation programs based on ACT. However, studies exploring the experience of health professionals in occupational rehabilitation have shown that they perceive improved quality of life as a better, or the principal, rehabilitation aim [29–31] and sometimes feel conflicted by having a RTW rehabilitation goal [31]. There have also been questions regarding whether a RTW agenda fits with the theory of cognitive behavioural therapies, to which ACT belongs, since a preconceived goal established by the therapists might endanger the therapeutic alliance and thereby the outcome of treatment [32]. In ACT, the therapist’s purpose is described as letting clients freely choose their own values, whatever they may be [33]. Both practice experiences and theoretical foundation thereby suggest that combining RTW as a declared goal of occupational rehabilitation with an ACT approach might be particularly challenging. Therefore, the aim of this study was to explore therapists’ experience of addressing the RTW process in an inpatient occupational rehabilitation program based on ACT.

## Methods

This was a qualitative study based on two distinct yet complementary data sources: interviews with therapists working with ACT-based occupational rehabilitation, and participant observation of the rehabilitation program. The combination of methods was chosen to explore the individual therapists’ experience and gain contextual insight into their practice at the rehabilitation centre. The interviews were analysed according to Interpretative Phenomenological Analysis (IPA), an approach informed by phenomenology, hermeneutics and ideography [34]. The study was conducted within the context of an ongoing randomized controlled trial [15].

### Study setting

#### Socio-cultural context

Norway is described as a ‘work-society’, and Norwegian welfare legislation has always placed great importance on the primacy of work [35]. However, the ‘work approach‘ introduced in the late 1980s represented a shift within the Norwegian welfare discourse; it explicitly linked benefits with work requirements by declaring that “the citizen has ‘a right and a duty’ to work or to prepare for work” [35], p. 11. After three days (or eight, in particular circumstances) of sick leave, employees must provide their employer with a sick note from their general practitioner (GP). Full wage compensation is given from day one; the employer covers the first two weeks, and the Norwegian Labour and Welfare Administration (NAV) covers the consecutive period up to 52 weeks. Workers can receive ‘work ability assessment benefit’ if their ability to work continues to be impaired after 52 weeks; however, the benefits are then reduced to two-thirds of the wages. It is the GP who treats illness or complaints and refers for further treatment or RTW rehabilitation if necessary [36].

#### Hysnes Rehabilitation Centre

Hysnes Rehabilitation Centre was established as part of St. Olav’s University Hospital to provide occupational rehabilitation. Persons living in one county in Central Norway (population 375,000) were eligible for inclusion if they were between 18 and 60 years old, had been on sick leave for 2–12 months, a current sick leave status of 50–100% and a diagnosis within the musculoskeletal, psychological or general and unspecified chapters of ICPC-2 (International Classification of Primary Care, Second edition) [15]. The program consisted of two stays, the first for 11 days and the second for five. In between, participants spent nine days at home engaging in an individualised therapeutic plan. The rehabilitation program was structured like a work day and included three entwined components: psychological treatment, physical training and work-related problem solving. The majority of the program (eight sessions, a total of 16 h) was provided in groups consisting of six to eight participants (regardless of diagnosis) and two therapists. These sessions followed a manual designed to cover key ACT processes and exercises. Participants were assigned to a primary therapist who also provided five individual sessions (a total of 5 h). In addition, the program consisted of seven mindfulness sessions (a total 3.5 h), ten individual/group-based supervised training sessions (a total of 12 h), six ‘walking to work’ sessions (a total of 3 h), and an outdoor activities day. Psychoeducational sessions covering stress, pain, sleep and nutrition were also provided, as well as one session specifically addressing work participation. The participants were given homework (e.g. reflecting on topics from the sessions, filling in worksheets and making plans) that was addressed in subsequent group or individual sessions. To include stakeholders, a midway and discharge summary and a RTW plan were sent to the participants’ GP, and to their employer and social security office if considered relevant and with the participant’s consent. In addition, participants were encouraged to invite relevant stakeholders such as family, friends, their GP, employer or co-workers to visit the rehabilitation centre on a network day. This day included two group sessions and a session with the participants’ primary therapist. All activities at the rehabilitation centre were conducted according to core principles in ACT, and all employees (including those working with exercise and in the kitchen) received guidance to ensure that a coherent program was provided. In addition, the therapists had team meetings where strategies concerning obstacles and possibilities for the participants’ rehabilitation process and RTW were discussed (a total of 5.5 h). A study describing participants‘ characteristics for a previous edition of the rehabilitation program (although with the same inclusion criteria) found that 77% were women, the mean age was 45 years, and 49% had higher education. At inclusion, length of sick leave was 224 calendar days, and the main diagnoses were dispersed as follows: 52% musculoskeletal, 38% psychological, and 10% general and unspecific [25].

#### Acceptance and Commitment Therapy (ACT)

The therapists worked according to a contextually adjusted model of ACT. The ACT model explains health and treatment, as well as pathology, through six interrelated and mutually dependent processes: 1) *contact with the present moment* instead of dominance of the conceptualised past and feared future, 2) *acceptance* instead of experimental avoidance, 3) *defusion* instead of cognitive fusion, 4) *self as context* instead of attachment to the conceptualised self, 5) *value clarity* instead of choices based on avoidance, social compliance, or fusion, and 6) *committed action* instead of inaction, impulsivity or avoidant persistency [17]. ACT is grounded within a framework of functional contextualism, and psychological events are described as “a set of ongoing interactions between whole organisms and historically and situationally defined context” [18], p. 646. The function and meaning of these events are addressed in treatment, not their presence or validity. Clients are encouraged to meet their thoughts and evaluations with openness and curiosity and “embrace a passionate and ongoing interest in how to live according to their values” [18], p. 647. The model further focuses on gaining understanding through experiential exercices, such as using metaphors and stories to directly come in contact with psychological processes, and is described as having a bottom-up, inductive nature [16]. The ideal therapeutic relationship is described as ranging from superficial and straightforward to intimate and profound as “it is not the form or topography of a particular relationship, but the fact that it functions to satisfy the goals and values of both participants of that relationship” [37], p. 8.

### Research participants and recruitment

#### Participant observation

Potential participants received written and oral information about the observation and signed a consent form before the participant observation started. Three therapists (one in training) and six participants took part in the therapy group where participant observation was conducted during the fall of 2015.

#### Interviews

The interviews were conducted between September 2015 and March 2016. The rehabilitation centre had some turnover of employees, and from a total of 15 therapists employed during this period, 11 were successively asked to take part in individual interviews. All those who were asked agreed to participate. The therapists received oral information about the study and signed an informed consent form prior to the interviews, and five women and six men between the ages of 26 and 62 participated. They had worked at the rehabilitation centre from three months up to six years, and their occupational and educational backgrounds were diverse, including fields like physiotherapy, psychology, nursing, preschool teaching, counselling and human movement science. Nevertheless, they all worked according to the ACT model and received monthly guidance from a certified ACT instructor. Sessions with participants were occasionally videotaped (with their consent) and revised with the instructor to further develop the therapists’ competence. Some also received additional supervision to become certified ACT instructors themselves. To ensure anonymity within a highly transparent field, the therapists’ characteristics are not presented individually.

### Data collection

#### Participant observation

Participant observation of the rehabilitation program was conducted by the first author (NEK), who has an MA in social anthropology, to gain insight into the rehabilitation program and the therapists’ practice. NEK participated actively in the program from a participant perspective, received the same number of individual sessions as regular participants, and took part in leisure-time activities. Field notes [38] were made during lectures and group sessions (when considered appropriate), between activities, and by the end of each day. The notes included descriptions of the area, the setting, topics of group sessions and instructions given, conversation topics, statements and nonverbal reactions, methodological considerations, analytic comments and diary notes concerning personal experiences of the rehabilitation. The researcher strived to uphold a naïve or curious stance and delineate between descriptions and personal interpretation (e.g., denoting silence and downwards gazes versus insecurity and discomfort).

#### Interviews

The first author (NEK) conducted the interviews with the therapists. They were conducted at the university, or on the premises at Hysnes Rehabilitation Centre, and lasted from 52 min to one hour and 27 min. The interviews were audiotaped and transcribed verbatim. While most IPA studies examine how interviewees make sense of important life experiences and often refer to psychological concepts [34], our approach was somewhat different, as we wanted to explore the therapists’ experience. To this extent, the topic of our study and the number of informants diverge somewhat from typical IPA studies. The interview guide predominantly consisted of questions intended to allow the therapists to elaborate on their experience of working with ACT as an approach to facilitate RTW, what they experienced to be the most meaningful aspects of their work, and how they related this to the rehabilitation program’s articulated aim of RTW. The therapists were also encouraged to provide examples of how they approached their participants. While several topics identified through the participant observation and past interviews were incorporated into the interview guide as interviews proceeded, the aforementioned questions remained constant.

### Data analysis

In line with the aim of exploring the therapists’ experience, the interviews constituted the main component of the present study. However, the analysis of field notes from the participant observation provided a broader understanding of the therapists’ practice that informed the subsequent analysis of interviews.

#### Analysis of field notes

The field notes were first read and then coded descriptively and conceptually. For example, descriptions of group sessions could include codes describing the setting and instructions given by the therapists (e.g., “there is no such thing as ‘the right’ way to feel”), as well as a further analytic interpretation (e.g., “experience as subjective” and “acceptance”). Codes were tentatively grouped into themes that informed the subsequent interview guides and revised during the analysis of the interviews. The totality of field notes was read by and discussed with MBR, who had also conducted participant observation during an earlier edition of the rehabilitation program. Since two themes identified as important from interviews were not exemplified by the therapists, data originating from field notes were included in the results to depict how these themes were communicated to participants.

#### Analysis of interviews

Interpretative Phenomenological Analysis was chosen as an analytic approach due to its ideographic commitment and recognition of several levels of abstraction throughout the analytic process [34]. The interviews were analysed successively according to the following procedure: First, the transcript was read while listening to the sound file, and corrections to language and sentiment were made. The transcript was then reread while consecutively making descriptive, linguistic, and/or conceptual comments. The comments were coded in NVivo [39] to systematise the content and further exported to Mindjet MindManager [40] to outline possible connections. The comments were then developed into emergent themes that were purposively kept closer to descriptive (e.g., “you cannot change your surroundings”) rather than conceptual comments (e.g., “limitations to agency”). These themes were further grouped and superordinate themes established. Next, quotations were added to form a summary table. If considered necessary, quotations were kept long to sustain the therapists’ line of reasoning. This procedure was repeated through all 11 interviews before the summary tables were combined. First, themes common to all interviews (such as “the values of work”) were extracted. Other themes were sorted according to their commonality or resemblance, and the titles were adjusted or abstracted to describe the content accurately. Some superordinate themes were occasionally split and renamed if the underlying meaning no longer reflected the new grouping. The joint summary table provided the frame for the first draft of the results; however, several themes were rearranged successively. The individual summary tables were continually revised during this process to ensure that the themes and analytic text reflected original sentiments and preserved individual differences. However, the therapists’ experiences are predominantly presented conjointly as their approaches and perspectives were surprisingly convergent. Quotations were included to illustrate, expand or add nuance to the themes identified. NEK performed the initial coding. However, MBR read all interviews, and the themes and analytic text were discussed and revised with MBR and NEK collaborating throughout the analytic process. MSF and RJ also contributed to the analytic process by reading two interviews each, participating in group discussions and commenting on drafts of the result section, resulting in some final adjustments (such as emphasising therapeutic relationship). The quotations were translated from Norwegian to English by NEK and revised by all authors to ensure that the original sentiments were maintained.

## Results

In the interviews, the therapists described the main goals and premises for their practice and how they sought to help participants move towards these goals through what we identified as processes concerning 1) ownership, 2) causes of sick leave, 3) relation to expectations, 4) the values of work, and 5) the scope of agency.

### The therapists’ goals and the premises for their practice

The therapists described the overarching goal of the rehabilitation as twofold: to support participants in building both a meaningful life and sustainable work participation for the rest of their occupational life. Neither of the goals were perceived as definite endpoints, but rather as processual activities to be maintained continuously throughout life. The therapists described the goals as closely intertwined and emphasised that it was necessary to approach the participants as whole persons, not just employees, to help them achieve sustainable work participation. Both ACT and the biopsychosocial model were described as larger frameworks for practice that allowed the therapists to address and normalise the interrelationship between body, thoughts, emotions and behaviour. While the biopsychosocial model was referred to as a general framework, ACT was described as the theoretical and practical approach that permeated and guided all aspects of the rehabilitation. The main objective of this approach, increasing psychological flexibility, was described as so comprehensive that it proved beneficial to all areas of life, including work participation. While the therapists sometimes emphasised different processes in the ACT model as important to participants’ RTW-process, no conflicting perspectives were found in the data material. However, several therapists described that the diversity in therapist and personal experience sometimes provided them with different perspectives and understandings of specific participants and that frequent discussions benefitted them, and in turn, the participants. In addition to ACT, the therapists frequently referred to research on how to facilitate RTW. They commonly stated that work is health promoting, and that the most beneficial approach to secure sustainable RTW is a quick return in a graded position with a progressive increase in work hours. However, the therapists emphasised that the RTW process is highly context-specific and had to be attended to individually.

### Processes of change

The therapists often referred to what they considered participants’ common challenges to move towards a meaningful life and sustainable work participation, and how they tried to facilitate this movement. We identified five processes the therapists attempted to instil: 1) enabling ownership of the process of change, 2) identifying the complex causes of sick leave, 3) changing how participants relate to own and others’ expectations, 4) unfolding the values of work, and 5) exploring the scope of agency. The therapists described how the changes they sought to facilitate were long-term processes that exceeded the timeframe of the rehabilitation program; they could only help instil them and enable participants to uphold them on their own. If not stated otherwise, the processes were attended to simultaneously throughout the rehabilitation program and did not follow a specific order.

**1) Enabling ownership of the process of change**

A recurring theme in all interviews was the necessity of enabling participants to take charge of and actively engage in their own rehabilitation process. Many therapists said that most participants initially expected to receive qualified advice and treatment that would improve their health situation and subsequently lead to work participation. They often sought external aid in their search for recovery as numerous previous attempts had failed. To counteract this passive search for a ‘quick-fix treatment’, the therapists said they let participants define their main challenges and personal agenda for the rehabilitation and refrained from providing advice.

The therapists all spoke of the importance of making the rehabilitation program personally meaningful to each participant; one way of ensuring this was to let them delineate their main challenges. According to the therapists, all participants found that their health problems had consequences for their life in general. If measured against RTW, the consequences concerning close relationships, such as family, were often perceived as more important. However, the majority of the therapists stated that addressing the topics chosen by the participant usually entailed simultaneously attending to their RTW process, as the underlying reasons for these difficulties often coincided with those causing sick leave. The therapists described the necessity of patiently building a trusting relationship with their participants by validating and normalising their experience, sometimes by sharing personal experience with the themes addressed. This was seen as a prerequisite for the participants to be open about their difficulties so the therapists could gain an understanding of their life situations, and later be able to address sensitive topics. Exploring and understanding the participants’ own assessments of their problems was referred to as a means to *finding the core of participants’ difficulties*, and was linked to being able to target the changes needed more effectively. A few therapists said that in some cases, they waited to address work participation until compiling the RTW plan towards the end of the program if the participants did not address it themselves. However, the therapists predominantly described that they gradually envisioned how the participants’ chosen topics were relevant for their RTW process.

There is nothing that cannot be talked about, and I believe this is new to many [participants]. “Wow! Can I talk about this? Can I be taken seriously and open up for things I have carried alone?” […] But after a couple of sessions, we start to address: “How does that affect my work situation?” and “what do you believe has to change for you to be able to stay at work, or return to work?” Because the pattern you have at home, you often have at work as well. There is a red thread increasingly leading towards the work situation, which, in the last session, results in a return to work plan. My sessions often concern the whole life [of the participant], and then we increasingly boil it down towards the work situation. – Phillip

The therapists further described how a key component of the rehabilitation program was to help participants move purposively towards what they considered meaningful in life, i.e. to live more in line with their personal values. Several therapists said that they refrained from presenting RTW as the sole agenda of the rehabilitation program, and rather attempted to show participants that they had their best interest in mind, regardless of their ability to work. The therapists described this as *having the same goal*, *playing on the same team,* or *sitting in the same boat*. Values were often likened to personal characteristics concerning whom you want to be and how you wish to be perceived, such as being a caring mother, being creative or contributing to something greater than yourself. Some therapists said that personal values did not necessarily coincide, and sometimes were at odds, with work participation. However, most therapists explained that they believed working towards a more meaningful life in general would create positive ripple effects in all areas of life and the likelihood of RTW would increase simultaneously. This was often referred to as the *transfer value* or *spread of effect*. Several therapists said that the participants’ closeness to resuming work had to be considered, and that prematurely introducing the topic of RTW was possibly counterproductive.

Sometimes it can be very advantageous to work solely on work participation, to push the topic of work. Then again, sometimes it is *dis*advantageous, and creates a lot of resistance. […] So, you often work with other life areas: relationships or self-care, health. You might work *a lot* in *those* areas. And then you have somewhat turned your back on the work situation. […] But then again, you know that you are working to increase quality of life. And if you increase quality of life, you most likely increase work ability as well. – Oscar

All the therapists explained that they strived to refrain from providing advice to spur the participants’ initiative to take charge of their own rehabilitation process and become more self-reliant. The therapists recognised that this could be uncomfortable for the participants since no alternative suggestions for action were presented, however, it was seen as a necessity to ensure that all new actions were initiated by the participant and experienced as personally meaningful.

They have tried so many things, things they have heard about from others, things they have read about, advice they have received. This is supposed to be more like something you have learned yourself, something you actually find important. That possibly makes it more persistent. Yes, something you truly believe in, that you have *experienced* works, or doesn’t work, or whatever it might be. I believe that’s the point. It’s more durable and more meaningful. – Nora

While the therapists said they believed not sharing their opinions was beneficial to the participants’ process, all expressed that they found it difficult or frustrating, especially if the participants’ perceptions diverged from their own. Some even said that they initially perceived it as counterintuitive to how they worked to help patients previously. Not providing advice was described as necessary to adhere to the ACT approach. The therapists explained that the alternative was to patiently pose numerous questions to help participants gain a new perspective of their situation and ask for their willingness to explore difficult thoughts and emotions as well as alternative actions. While acknowledging that this approach was more time consuming, the therapists believed it ensured greater commitment.

**2) Identifying the complex causes of sick leave**

A view commonly expressed by the therapists was that health problems often emerge and persist based on interrelated factors that, combined, affect health negatively. They said many participants perceived physical symptoms to be their main health challenge and believed these had to be significantly reduced before they could resume full work participation. While agreeing that physical symptoms were central to the difficulties participants experienced, the therapists said it was important to expand participants’ understanding of what caused symptoms to emerge, worsen and persist. Therefore, the therapists sought to help participants recognise how their symptoms were influenced by a complex mixture of burdens and by how they related to them.

The therapists all considered it important to help participants become aware of how the combination of burdens in their lives affected both health and work participation negatively. They explained that many elements in life other than work contributed to generating, sustaining and worsening symptoms participants experienced. A few added that being on sick leave only relieves one from duties related to work, not other areas in life. The therapists described helping participants identify challenges they experienced in all areas of life and investigating the connections between them. This was also addressed in an educational session on stress during the participant observation of the rehabilitation program, where the therapist used a metaphor of a glass filling up with water to exemplify how stressors accumulate over time and eventually spill over. He explained that while we have a natural tendency to identify the last occurrence as the reason for health problems to appear, the totality of stressors is often the cause.

The therapists also spoke of the need to make participants aware of how their health situations were influenced by the way they related to them. Several therapists stated that excessive focus on symptoms or arduous attempts to avoid them affects how symptoms are experienced in the present moment, and to what extent they are maintained. One therapist described this as a process of becoming increasingly aware of causation:

Pain is one thing; suffering is another. […] [Suffering] is all the collateral problems related to pain. The way you relate to it negatively affects all the areas it influences. It might contribute to sustaining some of the suffering instead of just refraining from that battle. […] Just realizing that often results in awareness of the problem and the cause. It’s a nice thing, that process of becoming aware and realizing how being affected by *being* in a conflict has resulted in chronic back pain because patterns, patterns, patterns have built up and problems have worsened. And you are tired because you fight against it all the time. So, I believe it is important to also work on that awareness. The experience of the cause and what has sustained it, often changes during those four weeks. – Oliver

The therapists often said that they aimed to help participants become aware of how patterns of thoughts, emotions and behaviours acquired throughout life influenced their current health situation. Several also spoke of the need to address participants’ relation to past experiences if they were considered to cause inappropriate patterns in the present. One therapist explained that he sometimes considered the relationship to traumas as the underlying cause of sick leave. While assuming that participants would possibly be able to resume work, he predicted that they would experience reoccurring sick leave if this relationship was left untreated. The therapists all described helping participants relate to thoughts and emotions as subjective assessments instead of representations of truth. One therapist stated that the common approach of the rehabilitation program was to expand participants’ view of their rehabilitation process by understanding thoughts, emotions and behaviour as an essential part of their health problems and recovery:

Those who come here, they come with their body. With pain, with exhaustion. And they say: “I am tired, and it is my body”. And then we say: “Rehabilitation is about something else. Rehabilitation has to do with the one who has that body, the one who has all these things. And that is why we start to talk about thoughts, emotions, behaviour, to start noticing *what happens when you are in contact with that body*”. Because this is often where the struggle takes place. […] And to start noticing yourself as an agent in relation to your body. What they have done has actually worked, until the body stopped. And they wish to continue the same way, but the body doesn’t allow it anymore. And then the question is: “What does that mean? Does it mean that you have a disease, or do you have to start relating differently to life?” – William

**3) Changing how participants relate to own and others’ expectations**

A recurring theme in the interviews was the importance of addressing the various expectations the participants experienced. The therapists described that many felt distressed by not being able to satisfy demands and expectations from society, their workplace, and close relations, but few had shared to what extent their health problems affected them. According to the therapists, many were hesitant to reveal information to their employers or colleagues, as they feared being perceived as vulnerable, having their health problems questioned or considered it private. While the participants often attributed expectations to their surroundings, the therapists considered them to frequently originate from participants themselves. To help participants relate more soundly to expectations, the therapists explained that they sought to help investigate the source of participants’ expectations and adjust them to a realistic level according to the current health situation.

The therapists said they wanted to help participants investigate the extent to which they themselves were generating and upholding the expectations that proved stressful. They acknowledged that all participants faced expectations from various sources; concerning work participation, several pointed to a general societal expectation of full employment and productivity. In addition, the majority used words such as *shame* and *stigma* to characterise the perception of long-term sick leave due to the diagnoses common to their participants. The therapists did, however, state that these perceptions influenced and shaped participants’ views of themselves, and mainly were present as internalised expectations acquired throughout life, some even instilled from childhood.

We see many parallels to upbringing. […] They bring with them this ‘productivity reflex’, that you should be productive all the time. […] And then they feel inadequate. But mother did it, grandmother did it, great grandmother did it. – Lucas

These internal expectations were considered potentially more harmful to participants’ health than expectations from others, and several therapists portrayed them as being unconsciously present and a constant driving force of action, one referring to them as an *inner whip*. Some also linked this to a tendency to assume that others have expectations, without having been told so directly.

In order to relate more soundly to various kinds of expectations, the therapists said they believed participants would profit from adjusting expectations according to their current health situation. One aspect of this was to help participants accept their current level of functioning; during the participant observation, this was explained by *the gap model* in an educational session on pain. The therapist drew two graphs on a whiteboard that gradually dispersed from one another to illustrate the increasing gap between the function participants tried to uphold and their true functional level. He then explained that this incoherence eventually leads to stress that influences health negatively and said that the ability to accept the current situation is crucial to lowering expectations of oneself to a realistic level.

The therapists further spoke of openness towards relevant others as either important or a prerequisite for reaching an alignment between level of functioning and expectations from others. Several therapists mentioned that participants needed to enforce their own limits, as no one else would do so for them, and that neither family, acquaintances, employers, nor colleagues could be expected to lower their expectations unless the participant explicitly communicated this need. The therapists also said they encouraged participants to expose their vulnerability instead of trying to uphold an image of themselves as strong and productive.

A lot of research shows that if you try to cover up vulnerability, hide it, then you spend a lot of energy on trying to be someone else. This concerns the participants’ own expectations as well as the employers’ expectations. […] It is hard to adjust expectations if the one you work with always portrays everything as okay. Then you are met with the same expectations. And you get frustrated because no adjustments are made. […] This leads to a downward spiral until everything stops. And then the employer is like: “Why did it stop? Wasn’t everything okay?” – William

While all the therapists said they encouraged participants to be open with employers and/or co-workers, several also emphasised that each participant had to delimit how much he or she wanted to share, to whom and when. They stated that ‘being open’ could be interpreted in several ways; one therapist added that the degree of openness had to be considered relative to each participant’s specific workplace:

You have to assess the individual context. “How is the dialogue with my employer or colleagues? What *is* the policy in our workplace?” You must figure out that for yourself because there are no explicit rules. […] I have experienced that they are not motivated for tears and openness at all in male-dominated occupations with a tough jargon. So obviously, being open might simply entail being *explicit*. – Emma

**4) Unfolding the values of work participation**

The therapists all explained that a central aspect of the RTW process was helping participants rediscover work participation as a personally meaningful activity. According to the therapists, almost all participants stated that they aspired to resume work and that work participation was very important to them. However, they predominantly referred to work participation in terms that suggested it to be a mandatory activity and an economic necessity. To expand this perception, the therapists said they attempted to help the participants unfold values connected either directly or indirectly to work participation and explore the connection to other areas of life.

The therapists described that unfolding values in all parts of life was essential to provide participants with a continuous direction that guided actions throughout life and led steadily towards a meaningful life and work participation. Exploring the values connected to the area of work was often portrayed as a perspective that was diametrically opposed to the participants’ focus on how health problems or external factors hindered work participation. A few therapists occasionally referred to work participation as a value in itself. However, the therapists predominantly considered work as a field or area where values could be attended to. They explained they sought to help participants explore what they found meaningful to their specific positions, or work participation in general, by posing different questions.

“Why do I go to work? What does my work give me? What can I contribute to at my work, and what can work contribute to for me?” And that is the colleagueship, the social interaction, to use your competency or the possibility to develop, to get paid, financial security, to contribute to something greater, like society. Be part of something bigger. Those things are often the values. […] Regardless of whether you are a mailman or a pilot, you will have some of those things. Community, pay, contributing, developing. And then the question is simply: “How does it play out in *your* life?” – Oscar

In addition, the therapists stated that they helped participants trace the link between work participation and other personal values. For example, the value of contributing to others could be attended to in the area of work, but also in everyday family life, by undertaking posts in organizations or engaging in voluntary work. Work participation could thereby be regarded as one of many activities that make it possible to continually fulfil personally meaningful values. This more indirect connection between values and work participation was considered particularly important in cases where participants did not experience their current work situations as meaningful. One therapist described how he helped participants discern how values connected to other activities could be brought into their work situation in the following manner:

Some of the time could, perhaps, be used to plan how you will find something that is meaningful and exciting, that which gives you something more. And you can find that when people start to talk about “I want to do that”, you can mirror it: “Now I see something happening to you!” Right? “Oh, I enjoy travelling”, “Okay! What do you enjoy about travelling?”, “Then I am optimistic, I am in a better mood, I cope better, I experience new things, I am curious, those kind of things”, “Okay, so you are curious and want to experience new things? Is it possible to bring that into the work situation, to give *that* some room?” – Oliver

The therapists generally described that unfolding the personal motivation for why one should work provided a stronger foundation to secure work participation than the normative assumption of having to work. Most of the therapists spoke of economic concerns as a poor incentive to uphold work participation. However, a few therapists said they sometimes adopted what they referred to as a *pragmatic approach*. At times, participants considered values that could not be realised through work participation as the most important in their lives, leaving the area of work irrelevant. One therapist explained that he would then acknowledge and address the economic necessity of work participation as a prerequisite for these values to be attended to:

You can always assume a more pragmatic approach. “What is most important in your life?”, “My family. Go hunting. Being outdoors two weeks a year”, “Okay, so work is not important?”, “No, I can have any kind of job, it doesn’t matter that much”. But for the *clear* majority, unless they wish to live a *very* ascetic life, in Norway, you *need* a job. If only to have the financial freedom to do the *other* things that are important to you. And as long as you perform well enough to keep that job, we can call that a daily goal: “Complete the workday so I can focus on what really matters to me. Tuesday: complete the workday so I can do what is important to me. Wednesday: the same”. – Lucas

**5) Exploring the scope of agency**

A recurring theme in the interviews was the importance of helping participants explore their true possibility of influence and encouraging them to take appropriate action. The therapists described that many participants came into rehabilitation believing that their surroundings and/or health situations had to change before they could resume work. According to the therapists, some participants undertook excessive activity in attempts to avoid difficult thoughts and emotions or change their work environment. In contrast, others remained passive, believing they had little influence. The therapists therefore considered it important to help participants explore limitations and possibilities of influencing various conditions and instigating actions coherent with personal values when appropriate.

The therapists all described that they aimed to help participants investigate whether they could influence and change a situation, or if doing would be a futile attempt inducing stress and further deteriorating their health. The therapists spoke of several external conditions as impossible to change or control, such as health and welfare services, the structural organization at work, or the personality of employer and colleagues. Internal conditions such as pain, exhaustion, thoughts and emotions were also identified as impossible to control through will. The therapists said they tried to help participants accept the circumstances they were not able to change. However, several emphasised that acceptance was a concept that could easily be misunderstood and thus had to be explained and treated cautiously.

I believe many connect ‘acceptance’ with resignation. It’s very important to address this when we use the concept of acceptance since many get provoked by it. And you have to use it respectfully, because it is not the same as resignation […]. You may refer to something as mundane as the weather: “I actually have to accept that it starts raining, I cannot change that. Or I can choose to spend a *lot* of energy. I can sit and talk about the rain all day, use all my energy on that. But is that what I want with my life?” That is one way of explaining acceptance. – Emma

The therapists further described that a key element of the rehabilitation program was to encourage participants to commence committed action regarding the situations they could influence and that ideally, these actions should be coherent with their personal values. Several emphasised that what they considered *proper* insight or understanding included the participants’ ability to practice the knowledge they developed during the rehabilitation program. The therapists all placed great importance on helping participants become aware of all the small choices they made during the day and question whether they actually had to do the things they perceived as mandatory. This was portrayed as diametrically opposed to unconsciously acting according to old patterns on autopilot, and was referred to as *breaking habits* and *patterns*, *getting out of the usual track* or *giving oneself more room to manoeuvre.* This availability of choice was described as liberating and empowering, but also frightening to some participants. One therapist pointed out that becoming aware of choices did not necessarily mean that participants chose different actions, but that it could lead to a feeling of assertiveness:

One of my recent participants said: “I have made a stop now and have felt that I actually have choices. And often I might make the same choice as before, but I feel that I made a conscious choice; it was not forced upon me by others”. And that became very important to her. I believe many acquire a perspective like: “I am at the helm. I drive this bus”. And that is not always easy, but at least there is more awareness of what you are doing. It doesn’t solely occur on impulse. You stop, check in on how you are feeling, and think it through. – Phillip

The therapists said they evaluated changes participants planned to make on the grounds of its feasibility to lead towards a meaningful life and work participation. While some participants suggested actions that the therapists considered appropriate, others did not. In these cases, the therapists described how they employed several approaches, but emphasised that they had to do so in a non-judgmental and curious manner to avoid creating resistance and to keep with the aim of enabling ownership. They explained that they initially attempted to help participants discover for themselves why their plans were inappropriate by posing questions intended to help participants become aware of how similar actions had not worked in the past or were incommensurate with their personal values. The therapists emphasised that not wanting to work or feeling unable to work could be a consequence of attempting to avoid difficult thoughts and emotions, since what is experienced as meaningful is not necessarily experienced as pleasurable. Several therapists spoke of their attempts to help participants differentiate between these emotions. Some did so by envisioning the ambivalence on a blackboard, others by encouraging participants to notice how thoughts, emotions and bodily sensations shape the experience of the present moment and asking if participants could make a conscious shift to act according to their values.

They get a tool, a specific tool: how to work on destructive thoughts and emotions, how to live with bodily symptoms that don’t disappear, to bring them along with you, and still work. I find that unique. Becoming aware of that choice can be a great change. They might hurt, have difficult thoughts and emotions, and still move in the direction they want. And many want to work. Becoming aware of that, wanting to contribute to society that way is important to many. That’s my experience, that the majority actually want to work. – Sarah

Several therapists described a feeling of being unsuccessful if participants nevertheless decided they were unable or did not want to resume work at the end of the program. However, they also said that they sometimes supported participants’ decisions to delay RTW, reduce their positions or resign to seek new employment if they considered it appropriate in a long-term perspective. While many therapists explained that they sometimes found it difficult not to follow up participants after the rehabilitation, several referred to their work as planting seeds that would continue to grow beyond the timespan of the program. Their role as therapists was thus to enable participants to continue their course autonomously.

## Discussion

The aim of this study was to explore therapists’ experiences of addressing the RTW process in an inpatient occupational rehabilitation program based on ACT. The results show that ACT was experienced as a meaningful approach to facilitate RTW, as it allowed the therapists to attend to all relevant aspects of the individual participant’s life that might influence work participation. The therapists described a twofold goal: to help participants create a meaningful life and engage in sustainable work participation. To do so, they attempted to instil long-term and interrelated processes concerning ownership, causes of sick leave, relation to expectations, values of work, and the scope of agency.

### RTW as a goal in rehabilitation based on ACT

The findings show that the therapists viewed the goals of creating a meaningful life and sustainable work participation as so closely entwined that they had to be approached simultaneously. Several studies have previously shown that health professionals find that it is difficult to have a RTW rehabilitation goal [31] and that improved quality of life is a better, or their principal, rehabilitation aim [29–31]. Consequently, RTW might become a secondary consequence of improved psychological and physical well-being [29, 30]. In the present study, the therapists predominantly described attending simultaneously to topics relevant to increasing quality of life and creating sustainable work participation. This reflects Hellman and colleagues’ view of the need to consider RTW as “a reciprocal process where well-being and work are interconnected and affect each other” [29], p. 503. ACT was often referred to as an approach that provided an opportunity to address all relevant aspects of the participants’ life that might influence RTW, which was seen as a necessity when facilitating work participation that was sustainable over time. Hence, the therapists’ approach could be described as comprehensive and multidimensional, since all aspects of life are considered relevant to the rehabilitation goal. Both the appearance and cessation of hindrances to RTW were described as a result of a complex network of interacting causes, resembling the concept of *systemic causation* [41]. In contrast with direct causation, systemic causation cannot be experienced directly since it often consists of multiple causes that, combined, or through feedback loops, have effects that are not traceable back to a single, identifiable cause [41]. The present study showed that the therapists viewed several topics, not necessarily perceived by the participants as connected to RTW (such as the relation to previous experiences and traumas), as necessary to address since they implicitly influenced RTW. Not explicitly addressing RTW was thus viewed as nevertheless attending to the RTW process by seeking out the ‘hidden’ causes of sick leave.

Having a framework for RTW that acknowledges the importance of addressing the full-life situation may be one of the reasons the therapists did not express difficulties with having a RTW rehabilitation goal. Their frequent reference to the compatibility between the ACT approach and RTW research also suggests that they engaged in a process of actively integrating the two goals. This may have been facilitated by the continuous guidance from a certified ACT therapist and interaction with a RTW research group evaluating the program. Further, the ACT approach appeared to unify the therapists in their interpretation of the causes of sick leave and solutions to RTW, as well as in their approach to participants. Based on the convergent descriptions provided in the interviews, the therapists seem to constitute a multidisciplinary rehabilitation team that functions as a coherent whole by being oriented towards an integrated (yet twofold) outcome instead of a mere collection of discipline-oriented therapists [42]. While the therapists did point to inter-therapist discussions that were not accessible through the methods and positioning chosen in this study, these discussions were described as valued contributions to practice, not diverging perspectives on how to attend to RTW. It is, however, reasonable to question whether participants might perceive the goal of sustainable work participation as less significant if RTW was only implicitly attended to during large parts of the rehabilitation program. This should be investigated in future studies.

### The necessity and challenge of including participants in rehabilitation

The therapists’ overall approach to participants also revealed a paradox within the context of occupational rehabilitation practice; not addressing RTW is sometimes perceived as the best approach to facilitate it. This perspective is in line with the learning/changing frame of reference described by Eikenaar and colleagues [43] in their study of the tacit and normative dimensions that organise and inform professionals’ interaction with clients. Within this frame, professionals believe that clients’ efforts should originate from autonomous conviction instead of external incentives. Rather than caring *for* clients, professionals resume a more passive role, emphasising the client’s own responsibility and capacity and attempting to learn them to become autonomous [43]. This approach is also in line with how literature on ACT describes the therapist’s purpose, namely to let clients freely choose their values, and help them “contact naturally occurring reinforcement for living consistent with his or her chosen values, whatever they may be” [33], p. 95. Eikenaar and colleagues [43] further connect the learning/changing frame of reference to an empowerment perspective which is also mirrored in the therapists’ acknowledgement of participants as better suited to finding solutions to their difficulties in the present study. However, this empowerment perspective also proves challenging to therapists who need to ensure participants’ right to self-determination while simultaneously having their own intellectual agenda, also referred to as “an act of balancing on a slack rope” [44], p. 229. All the therapists in the present study voiced this challenge. Refraining from providing advice was predominantly described as meaningful since plans would be more relevant and feasible if the participant was active in developing them. However, the therapists were frustrated when participants planned actions they considered inappropriate, and described several approaches intended to nevertheless lead participants towards what might create sustainable work participation. While not providing advice was attributed to the ACT approach specifically, the shift from therapists’ authoritative position to the users right to influence is described as an important objective in Western welfare states in general [44]. Nevertheless, the findings of the present study suggest that the therapists granted participants what can be termed a particularly high degree of influence.

The therapists approached their participants’ RTW process by letting them delimit their main challenges and set their own agendas to a large extent, without confining them to work-related topics. This is also in line with the proposition that therapists’ new role within an empowerment perspective demands certain skills and attitudes, including ‘hermeneutic competence’, i.e. the ability to be aware of how users experience and interpret their situations [44]. A similar view is described by Coutu and colleagues [45], who underline the importance of identifying and acknowledging patients’ representations of health and illness to facilitate RTW. Representations are described as thoughts, beliefs and attitudes developed through social interaction, experiences of being ill, and models of thinking, knowledge and information acquired from education, media and informal learning [45]. The five processes in the present study provide an example of how such representations can be included and attended to in the rehabilitation process. The therapists described a process of aligning and increasing awareness of any representations that led to actions which conflicted with the therapeutic approach and the stated aim of the rehabilitation (RTW). As such, the representations were not limited to health and illness as described by Coutu and colleagues [45], but expanded to include any representation that might hinder a meaningful life and sustainable work participation.

### The meaning of work participation

The findings show that the therapists addressed RTW explicitly through the process of unfolding personal values connected to work. Participants were encouraged to investigate how work participation could be experienced as a personally meaningful activity, not solely a normative expectancy of fulltime employment. The meaning of work has previously been defined according to the following components: 1) significance, 2) orientation, and 3) coherence [46]. The presence of significance is further described as “the value of work in the subject’s view, and his definition or representation of it” [46], p. 4, and concerns the importance attributed to work and potential interference with other valued life domains [47]. Others have also pointed out that long-term sick leave entails a process of redefining life priorities where other identities are given importance in the absence of work [48, 49], which in turn might influence RTW. Similarly, the study by Rise and colleagues [28] describes how participants found that an ACT-based rehabilitation program provided them with increased awareness of what they found important in life, and that they needed to balance work participation with other parts of life. This suggests a perception of work as interconnected with other parts of life, but that efforts spent in one area must be detracted from those spent in another. Literature on the work-life/work-family interface is often dominated by such a conflict perspective, emphasizing how the attempt to fulfil multiple roles creates stress and impairs well-being [50]. Nevertheless, this balance-oriented interpretation has been criticised by Thompson and Bunderson [51] for becoming a zero-sum time allocation exercise that “misrepresents the complex psychological processes by which people make sense of their time and manage multiple life domains” [51], p. 18. Instead, they propose that the quality and meaning of time spent on different activities creates a spillover relationship between work and non-work, characterised by complementary and synergistic interaction instead of competition. The findings of the present study seem to coincide with this perspective as personal values are seen as surpassing, and thereby connecting, different areas of life. While different burdens are perceived as collectively impairing health, living according to one’s values is portrayed as having a similar, but positive, spillover effect on health. Work participation is thus viewed as an activity infused with meaning which is interwoven with other areas of life. Whether the therapists’ intention is transferred to participants is uncertain and should be investigated further.

In the present study, the therapists dismissed economic necessity and normative expectations as the ideal motivation for RTW. This can be further explored by looking to the Norwegian context. Norway is known for having a generous social security system, making economic necessity a weak incentive for RTW before the one-year limit of full compensation. To this extent, it seems appropriate to approach RTW in terms of the personal meaning work might entail for an individual. However, the shift from ‘welfare’ to ‘workfare’ in the last decades is described as replacing passive support with work requirements, thereby using sticks rather than carrots to strengthen incentives to work [35]. This is also mirrored in ongoing discussions in Norway regarding welfare benefits and sick leave which often include moral arguments concerning the duty to work and to be self-sufficient. Particularly, groups without visible disabilities are reported to be treated with suspicion and have their willingness to work questioned [52]. Thus, the Norwegian discourse reflects what has been described as the new work ethic for our time where “full citizenship and personal fulfilment are only attainable through participation in paid labour” [53], p. 223. In the present study, the therapists’ approach to work may at first glance appear to be highly individualistic, disregarding societal obligations in favour of personal values and autonomous conviction. However, the therapists described actively initiating several approaches if maintaining participants’ autonomy led to plans or action considered not suitable, which is far from assuming the passive role previously described in the learning/changing frame of reference [43]. The therapists described that they generally encouraged participants to resume work at a slow pace, and gradually increase work participation to ensure sustainability. They also emphasised the importance of maintaining contact with the workplace to avoid alienation and alternating between full presence and full absence, all in line with the Norwegian Inclusive Work Life agreement of 2014–2018 [12]. The therapists’ description of assuming a pragmatic approach and their emphasis on initiating committed action despite thoughts and feelings of discomfort show that RTW is not reduced to a question of personal fulfilment or motivation.

It is however worth noting that while the therapists describe a systemic perspective when explaining how they understand the causes of sick leave and solutions to RTW, they simultaneously describe an individualistic perspective when delimiting the scope of their own influence. Here, they emphasise that they only have the opportunity to work with individual participants, make them self-reliant and enable them to take further charge of their RTW process. This individualistic/empowerment-oriented perspective reflects their theoretical commitment to ACT as well as the difficulty that arises in practice when attempting to account for the complexity and range of what might affect RTW. The therapists explain that it is important to delimit the participants’ scope of agency and thereby reduce the stress that might emerge from trying to change what is outside participants’ control. However, it is reasonable to question whether the therapists simultaneously risk psychologising and/or individualising the causes of sick leave and solutions to RTW by emphasising participants’ *relation to* challenges, a concern also voiced regarding mindfulness, another third wave cognitive behavioural therapy [54]. This might obscure the extent to which RTW could be dependent on changing unhealthy working conditions (e.g. conflicts, organization of work, increased workloads) or structural elements (e.g. employability in the labour market). RTW is now recognized as a multi-stakeholder process, and including the workplace in rehabilitation has proven effective when facilitating RTW [55–58]. The network day of the rehabilitation program in the present study provided such an opportunity for employers/co-workers to attend, if invited by the participant. If not, the workplace was left extraneous to the RTW process, only passively receiving information afterwards through the RTW plan. Future rehabilitation programs should include active involvement of the workplace/employers. In addition, therapists should pay close attention to how participants come to understand their roles and responsibilities in the RTW process.

### Strengths and limitations

The lack of previous research on this topic necessitated a broad exploration, and we chose a combination of interviews and participant observation to investigate the aim of the study. These methods made it possible to explore therapists’ individual experiences in interviews and acquire contextual knowledge of their practice by partaking in one rehabilitation program. The participant observation was constructive to several aspects of the study; it provided significant information that informed the interview guide and facilitated interviews and it was particularly important to the analytical process. The article was the collaborative effort of four authors with different backgrounds. The analysis was primarily conducted by two authors with backgrounds in social science/public health. The two other authors, who had medical and/or public health research backgrounds, read selected interviews, discussed the analysis and commented on drafts of the article. To further strengthen the interpretations and affirm the results, the analysis and preliminary results were presented and discussed in an inter-professional research group on several occasions. There are also some limitations to the study. The therapists’ perceptions were surprisingly coherent, making it challenging to elicit differences. Possible discrepancies or disagreements might have been detected through focus-group discussions [59] or by observation of team meetings. As the participant observation of the rehabilitation was performed from a participant perspective, the researcher did not have access to such settings (or sessions where RTW was attended to individually). Observations could have elicited additional information important to the aim of the study, and future research should seek to include (participant) observation of multiple settings. It should however be noted that this method also presents some limitations. The researcher’s extended role necessitates increased reflexivity regarding the role subjectivity plays in both the field setting and the interpretative process, especially since the aim of this study was to explore the therapists’ experiences, not their interactions with participants. Preparations before conducting the fieldwork, continuous reflective notes regarding positioning and interpretation, consideration of the representativeness of field data, triangulation of methods, and inclusion of several researchers in the analytical process were efforts taken to attend to these limitations. Further, the unique socio-cultural context limits the transferability of the results. Characteristics of Norwegian legislation and rehabilitation practice include full economic compensation up to one year of sick leave, the possibility and encouragement of graded sick leave, and in this case, a 3.5-week inpatient occupational rehabilitation program. The generous welfare system probably provides a context more compatible with certain aspects of ACT (e.g. exploring why engaging in paid labour is personally meaningful) than compensation systems found elsewhere. Another limitation of this study is that characteristics of the participants at the rehabilitation centre necessarily shape how the therapists approach RTW. However, the therapists’ experiences should not be seen as completely determined by the specific context at hand. We therefore believe this study provides knowledge on how therapists may address the RTW process of sick-listed participants within an ACT approach and the potential dilemmas they need to manage while doing so.

## Conclusion

This qualitative study shows that the therapists experienced addressing the RTW process by instilling several processes that should jointly lead towards a meaningful life and sustainable work participation. The ACT approach was described as meaningful and appropriate in facilitating RTW since it allowed therapists to attend to the complexity of work disability and RTW, help participants rediscover important values connected to work, and strengthen ownership of their RTW process. The unfolding of personal values might integrate work participation with other areas of life and thereby reconcile the tension between work and family life. Providing work participation with personal meaning as a motivation for RTW also seems especially commensurable with the legislative context of Norway, where economy presents a poor incentive for RTW. However, this suggests that applicability might be limited as well. This study also exposes other potential theoretical and practice-related challenges when working with ACT in the context of RTW rehabilitation that therapists should be attentive to in order to secure the prominence of RTW. Expanding the scope of what might influence RTW and letting participants delimit the agenda of rehabilitation without confining it to RTW increases the need to relate the themes addressed explicitly to the participants’ RTW process. Therapists should also be aware of the dilemma that potentially arises when they attempt to refrain from providing advice while simultaneously encouraging actions they consider appropriate to facilitate sustainable work participation. The individualistic/empowerment-oriented perspective therapists describe may also obscure the extent to which RTW is a multi-stakeholder process, and therapists should pay close attention to how participants come to understand their roles and responsibilities in the RTW process.

Future qualitative research should investigate how participants’ experience that ACT-based rehabilitation influences their RTW process and quality of life, and to what extent the therapists’ intentions are reflected in their experiences. The use of participant observation could provide knowledge of the interactional and behavioural aspects of the RTW process. Studies from different socio-cultural contexts might provide important information on the appropriateness of using ACT in different RTW rehabilitation settings.

## Abbreviations

ACT: Acceptance and Commitment Therapy
GP: General Practitioner
IPA: Interpretative Phenomenological Analysis
RTW: Return to work
